# Prognostic Value of the Combined Lymphocyte-to-Monocyte Ratio and Handgrip Strength in Patients with Resected Pancreatic Head Cancer

**DOI:** 10.3390/cancers18142227

**Published:** 2026-07-10

**Authors:** Kazushi Yamashita, Daisuke Suzuki, Katsunori Furukawa, Tsukasa Takayashiki, Satoshi Kuboki, Shigetsugu Takano, Masayuki Ohtsuka

**Affiliations:** 1 Department of General Surgery, Graduate School of Medicine, Chiba University, Chiba 260-8670, Japan; y.kazushi1001@gmail.com (K.Y.); takayashiki@hospital.chiba-u.jp (T.T.); satoshi.kuboki@faculty.chiba-u.jp (S.K.); stakano@faculty.chiba-u.jp (S.T.); otsuka-m@faculty.chiba-u.jp (M.O.); 2 Department of Surgery, Teikyo University Chiba Medical Center, Ichihara 299-0111, Chiba, Japan; 3 Department of Surgery, National Hospital Organization Chiba Medical Center, Chiba 260-8606, Japan; katsunorifurukawa3948@gmail.com

**Keywords:** handgrip strength, sarcopenia, lymphocyte-to-monocyte ratio, inflammation-based prognostic score, pancreatic head cancer, pancreaticoduodenectomy

## Abstract

Background/Objectives: The lymphocyte-to-monocyte ratio (LMR) is an inflammation-based prognostic score (IBPS) that can be easily calculated using routine blood tests. Handgrip strength (HGS), a key diagnostic component of sarcopenia, also predicts survival. Nonetheless, the prognostic role of combined LMR and HGS in patients with pancreatic head cancer undergoing pancreaticoduodenectomy remains unclear. This exploratory study aimed to analyze whether the combination of preoperative LMR and HGS is associated with overall survival. Methods: We retrospectively analyzed 105 patients with pancreatic head cancer who underwent pancreaticoduodenectomy at Chiba University Hospital from January 2016 to December 2020. We examined the prognostic values of IBPSs and HGS and compared combinations of preoperatively measurable factors. Results: Multivariate analysis assessing preoperatively measurable factors demonstrated that low preoperative HGS, low preoperative LMR, and high preoperative carbohydrate antigen 19-9 level were associated with poor overall survival (hazard ratio [HR]: 2.41, 95% confidence interval [CI]: 1.29–4.51, *p* = 0.006; HR: 2.05, 95% CI: 1.12–3.76, *p* = 0.020; and HR: 2.68, 95% CI: 1.25–5.76, *p* = 0.011, respectively). The combination of low HGS and low LMR was associated with poor overall survival, although only 12 patients were included in this subgroup. Multivariate analysis evaluating clinicopathological factors demonstrated that this combination was associated with poor overall survival (HR: 2.85, 95% CI: 1.06–7.69, *p* = 0.038). Conclusions: In this single-center exploratory study, the combination of preoperative LMR and HGS was associated with poor overall survival in patients with surgically resected pancreatic head cancer. Further prospective multicenter validation is required before this combined marker can be used for clinical decision-making.

## Simple Summary

Pancreatic cancer has a poor prognosis, and pancreaticoduodenectomy is one of the most invasive procedures in gastrointestinal surgery. Therefore, preoperative prognostic assessment is important for selecting appropriate surgical candidates and planning perioperative management. Although several prognostic factors for pancreatic cancer have been reported, many are based on pathological findings that can only be evaluated after surgery. Thus, simple and clinically applicable preoperative prognostic markers are needed. In this exploratory single-center study, we investigated the prognostic value of combining the lymphocyte-to-monocyte ratio, an inflammation-based prognostic score, with handgrip strength in patients with pancreatic head cancer undergoing pancreaticoduodenectomy. Our findings suggest that the combination of the preoperative lymphocyte-to-monocyte ratio and handgrip strength may help identify high-risk patients before surgery, but these results should be validated in larger multicenter cohorts before clinical application.

## 1. Introduction

The inflammation-based prognostic score (IBPS), which can be easily calculated from blood biochemical tests, is a useful prognostic factor in various cancers [1,2,3,4,5,6]. IBPSs, such as lymphocyte-to-monocyte ratio (LMR), neutrophil-to-lymphocyte ratio (NLR), and platelet-to-lymphocyte ratio (PLR), are prognostic gastrointestinal cancer markers [6,7,8]. Recently, Okugawa et al. revealed the usefulness of the lymphocyte-C-reactive protein ratio (LCR) as a prognostic marker [9].

The European Working Group on Sarcopenia in Older People (EWGSOP1) defined the diagnostic criteria for sarcopenia in 2010 [10]. This definition was subsequently revised by EWGSOP2 in 2018 [11]. Sarcopenia is associated with poor prognosis in various cancers [12,13,14]; nevertheless, its diagnosis requires the measurement of muscle mass. Handgrip strength (HGS), a marker of muscle strength used in the diagnosis of sarcopenia, is useful for preoperative prognostic prediction. HGS predicts survival in gastric cancer and liver cirrhosis [15,16].

Pancreatic cancer has a remarkably poor prognosis; the median survival after radical resection for patients with pancreatic cancer is 21–28 months [17,18]. Pathological factors, such as tumor size, histologic grade, and distant metastasis, as well as serum carbohydrate antigen (CA) 19-9 levels, are routinely used to predict prognosis in pancreatic cancer [17,19]. However, these well-established prognostic factors are dependent on histological examination and can only be assessed postoperatively.

More recently, clinical staging systems for localized pancreatic ductal adenocarcinoma have emphasized the importance of preoperatively available factors. For example, the Trans-Atlantic Pancreatic Surgery (TAPS) Consortium proposed an ABC staging system based on tumor anatomy, CA19-9, and performance status at diagnosis [20]. These approaches highlight the need for simple preoperative markers that capture not only tumor-related factors but also host-related vulnerability. In this context, the combination of HGS and LMR may provide complementary information by reflecting both physical reserve and systemic inflammatory or immune status.

IBPSs, such as LMR, NLR, and PLR, have important roles in predicting pancreatic cancer survival before surgery [5,21]. Nonetheless, the prognostic role of IBPSs in patients with pancreatic head cancer undergoing pancreaticoduodenectomy (PD) remains unclear.

Patients with pancreatic head cancer and sarcopenia exhibit a poorer prognosis than those without sarcopenia [14]. However, few studies have investigated the prognostic role of HGS in pancreatic head cancer. In pancreatic head cancer, preoperative prognostic prediction is important, but few clinically applicable prognostic markers are available. Moreover, PD is one of the most invasive procedures in gastrointestinal surgery [22]. Therefore, preoperative prognostic assessment is crucial for determining surgical indications.

In this study, we aimed to evaluate a preoperative prognostic marker for pancreatic head cancer. We reexamined various IBPSs as prognostic markers and investigated whether combining IBPSs with HGS is more useful than either alone as a simple prognostic marker in patients with pancreatic head cancer undergoing PD.

## 2. Materials and Methods

### 2.1. Study Population

We retrospectively analyzed the prognosis of 105 patients with pancreatic head cancer who underwent PD at Chiba University Hospital from January 2016 to December 2020 and for whom preoperative HGS data were available. This study was designed in accordance with the Declaration of Helsinki. The appropriate ethics committee approved the study. This study was registered with UMIN-CTR (UMIN 000049450). Because this was a retrospective study, the requirement for written patient consent was waived.

### 2.2. Measurement of HGS

Preoperative HGS was measured within 7 days before surgery during the preoperative hospitalization period. HGS was measured twice on each hand with the patient standing, and the maximum value was used for analysis. Grip strength was measured primarily using a digital hand dynamometer (Grip-D, T.K.K. 5401; Takei Scientific Instruments Co., Ltd., Niigata, Japan). To measure HGS, the dynamometer was held and adjusted so that the second joint of the index finger was at 90°. Measurement was performed with the arm naturally lowered at the side. Measurements were performed by trained medical staff according to the institutional protocol. Formal interobserver or intraobserver reliability testing was not performed because of the retrospective nature of this study, and this issue is acknowledged as a limitation.

### 2.3. Data Collection

Each patient’s blood sample was collected as part of routine preoperative laboratory testing within 1 week before surgical resection, during preoperative admission. The blood samples were processed immediately in the institutional clinical laboratory according to standard procedures, and no stored serum or plasma samples were used for the calculation of IBPSs. We calculated preoperative IBPSs (LMR, NLR, LCR, albumin-to-globulin ratio [AGR], PLR, C-reactive protein-to-albumin ratio [CAR], advanced lung cancer inflammation index [ALI] score, systemic inflammation response index [SIRI] score, and systemic immune-inflammation index [SII] score).

Blood samples were collected in the morning after overnight fasting. As only routine clinical laboratory data were used in this study, storage conditions are not applicable.

Skeletal muscle mass index (SMI) was calculated by dividing the skeletal muscle mass area (SMA) by the square of the height (cm^2^/m^2^). SMA was quantified by analyzing contrast-enhanced CT cross-sectional images at the third lumbar vertebral level and measuring the skeletal muscle area.

### 2.4. Postoperative Follow-Up for Patients with Pancreatic Head Cancer

Patients with pancreatic head cancer received postoperative adjuvant chemotherapy with S-1 for 6 months when tolerated. The initial S-1 dose was determined according to body surface area and renal function based on the institutional protocol. Dose reduction, temporary interruption, or discontinuation was determined by the treating physician according to adverse events, patient tolerance, organ function, and performance status. If the patient refused or was deemed intolerant to adjuvant chemotherapy by the treating physician, no adjuvant chemotherapy was provided, and the patient was followed up for observation only. Postoperative follow-up included medical examinations, multidetector row computed tomography, tumor marker assessment, and other laboratory tests. Magnetic resonance imaging and other tests were also performed as required. Overall survival was calculated from the date of surgery to the date of death or the date of last confirmed follow-up. Patients who were alive at the last follow-up were censored.

The dose of S-1 was determined according to body surface area (BSA): 80 mg/day for patients with a BSA of <1.25 m^2^, 100 mg/day for those with a BSA of ≥1.25 m^2^ and <1.5 m^2^, and 120 mg/day for those with a BSA of ≥1.5 m^2^. If protocol-defined adverse events occurred during treatment, S-1 was temporarily discontinued. After recovery to the predefined criteria for treatment resumption, therapy was resumed with a one-level dose reduction.

### 2.5. Statistical Analyses

Continuous variables are presented as median (interquartile range) or mean ± standard deviation, as appropriate, and were analyzed using the Mann–Whitney U test or t-test, as appropriate. Categorical variables are expressed as numbers/percentages and were analyzed using the chi-squared or Fisher’s exact test, as appropriate. Overall survival (OS) time was calculated from the date of surgery to either the date of the last hospital visit or the date of the patient’s death. OS is presented as Kaplan–Meier curves, and differences between groups were analyzed using the log-rank test. Patients who were alive at the last follow-up were censored.

Optimal cutoff values were determined by receiver operating characteristic (ROC) curve analysis and the area under the curve (AUC). The point on the ROC curve where both sensitivity and specificity were maximized was selected as the optimal cutoff value. Because the cutoff values were derived from the same cohort used for survival analysis, the possibility of overfitting could not be excluded. Therefore, the cutoff values and the prognostic performance of the combined marker were interpreted as exploratory.

Sensitivity analyses using previously reported LMR cutoff values were performed when applicable. In addition, because several inflammation-based markers, including LMR, NLR, PLR, SII, SIRI, and related indices, are calculated from overlapping blood cell components, potential collinearity was considered. These markers were not forced simultaneously into the same multivariable model. Correlations among these biomarkers were assessed using Spearman’s rank correlation coefficient. The results of the correlation analyses and sensitivity analyses are presented in Supplementary Tables S1 and S2, respectively.

ROC curves were also used to assess the predictive value of prognostic factors for survival, and multiple logistic regression was used to identify independent factors associated with OS. AUCs were calculated to assess the ability of each variable to discriminate OS. In addition to the asymptotic 95% confidence intervals (CIs), *p* values under the null hypothesis (true area = 0.50) were also determined.

Multivariate analysis using the Cox proportional hazards model was performed with variables that showed significant differences in univariate analysis and were selected using a stepwise method (*p* = 0.05 for inclusion and removal). To evaluate whether HGS and LMR had a statistical interaction, an exploratory interaction analysis was performed by adding an HGS × LMR interaction term to the Cox proportional hazards model. *p* values < 0.05 were considered statistically significant. All statistical analyses were performed using JMP Pro version 15.1.0 (SAS Institute, Cary, NC, USA).

## 3. Results

### 3.1. Cutoff Values

The median follow-up period was 22.2 months. ROC curve analysis, with death as the response variable, was used to estimate the HGS cutoff value separately for male and female patients. The cutoff values for HGS were 29.0 and 21.0 kg for male and female patients, respectively. Because these cutoff values were derived from the present cohort, subsequent analyses were interpreted as exploratory.

The optimal cutoff values for IBPSs, CA19-9, and gait speed were calculated (LMR: 3.13, NLR: 5.3, LCR: 3820, AGR: 3.1, ALI: 0.05, PLR: 198, CAR: 0.02, SIRI: 1652, SII: 1318, CA19-9: 625, gait speed: 1.44 [m/s]).

The cutoff values for the body mass index in males and females were 20.9 and 20.1 kg/m^2^, respectively. The cutoff values for the SMI in males and females were 39.7 and 37.3 cm^2^/m^2^, respectively.

### 3.2. Relationship Between HGS and LMR and Prognosis of Pancreatic Head Cancer

**Figure 1:**
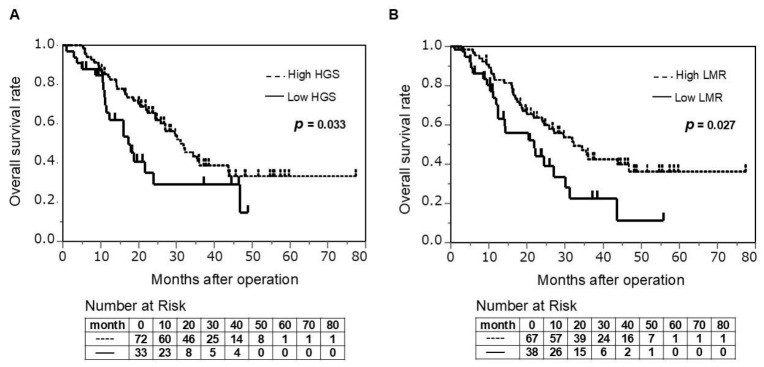
(**A**): Overall survival curves for patients with low versus high HGS. (**B**): Overall survival curves for patients with low versus high LMR. LMR: lymphocyte-to-monocyte ratio; HGS: handgrip strength.

OS was significantly worse in the low HGS group than in the high HGS group (*p* = 0.033) (Figure 1A). Moreover, it was significantly worse in the low LMR group than in the high LMR group (*p* = 0.027) (Figure 1B).

Univariate analysis assessing preoperatively measurable factors demonstrated that low preoperative HGS, low preoperative LMR, and high preoperative CA19-9 level were risk factors for poor OS. Additionally, these factors were independent predictors of poor OS in multivariate analysis (Table 1).

### 3.3. Comparison of Factors Associated with OS

**Figure 2:**
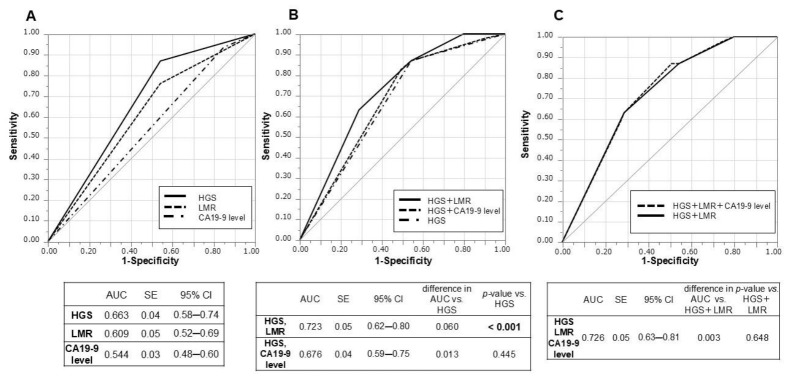
ROC analysis comparing the prognostic performance of HGS, LMR, and CA19-9 levels in patients with pancreatic head cancer undergoing PD. (**A**): Prognostic performance of HGS, LMR, and CA19-9 levels. (**B**): Comparison of HGS, HGS + LMR, and HGS + CA19-9 level. (**C**): Comparison of HGS + LMR and HGS + LMR + CA19-9 level. The *x*-axis indicates 1-specificity, and the *y*-axis indicates sensitivity. LMR: lymphocyte-to-monocyte ratio; HGS: handgrip strength; CA19-9, carbohydrate antigen 19-9.

We analyzed three key preoperatively measurable factors (HGS, LMR, and CA19-9 level) and compared their prognostic performance in patients with pancreatic head cancer undergoing PD (Figure 2). HGS showed a higher AUC for predicting OS than LMR and CA19-9 level in this cohort (Figure 2A).

ROC curves were generated using HGS, LMR, and CA19-9 levels to compare their prognostic performance. We assessed statistical differences in AUC values for OS among HGS + LMR, HGS + CA19-9 level, and HGS alone. In this cohort, the AUC was higher for HGS + LMR than for HGS alone or HGS + CA19-9 level (Figure 2B).

We also evaluated statistical differences in AUC values between HGS + LMR + CA19-9 level and HGS + LMR. Adding CA19-9 to HGS + LMR did not clearly improve prognostic discrimination in this cohort (Figure 2C). However, because CA19-9 is an established prognostic marker in pancreatic cancer and the present study had a limited sample size, this finding should be interpreted cautiously.

Based on these exploratory findings, we focused on HGS + LMR to further evaluate its clinical impact as a simple composite marker reflecting both host physical reserve and systemic inflammatory/immune status in patients with pancreatic head cancer undergoing PD.

### 3.4. Relationship Between Low HGS + Low LMR and Prognosis of Pancreatic Head Cancer

**Figure 3:**
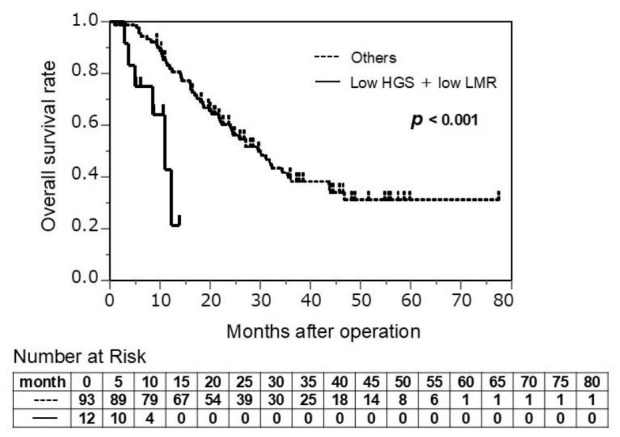
Overall survival curves for patients with low HGS + low LMR versus other patients. LMR: lymphocyte-to-monocyte ratio; HGS: handgrip strength.

OS was significantly worse in the low HGS + low LMR group than in the other groups (*p* < 0.001) (Figure 3).

Univariate analysis assessing clinicopathological factors showed that low preoperative HGS + low preoperative LMR, combined vascular resection, venous invasion grade 2 or 3, lymph node metastasis, non-R0 resection, high preoperative CA19-9 level, and non-completion of adjuvant chemotherapy were risk factors for poor OS. Multivariate analysis showed that low preoperative HGS + low preoperative LMR, combined vascular resection, venous invasion grade 2 or 3, lymph node metastasis, and non-completion of adjuvant chemotherapy were independent risk factors for poor OS (Table 2). Because only 12 patients were included in the low HGS + low LMR group, the hazard ratio estimate should be interpreted cautiously.

Exploratory interaction analysis was performed by adding an interaction term (HGS × LMR) to the final multivariable Cox proportional hazards model. The interaction term was not statistically significant (*p* for interaction = 0.505), suggesting that the prognostic value of combining HGS and LMR reflects a composite clinical marker rather than a statistically significant interaction between the two variables.

Sensitivity analyses using two previously reported LMR cutoff values (3.0 and 2.86) yielded consistent findings. The low HGS + low LMR group remained an independent predictor of poor overall survival using both the 3.0 cutoff (HR 1.91, 95% CI 1.04–3.50, *p* = 0.044) and the 2.86 cutoff (HR 2.00, 95% CI 1.01–3.69, *p* = 0.032) (Supplementary Table S2).

### 3.5. Characteristics of Patients with Pancreatic Head Cancer Undergoing PD

The characteristics of patients with pancreatic head cancer undergoing PD are shown in Table 3. In our study population, 12 of the 105 patients (11%) were classified into the low HGS + low LMR group. The low HGS + low LMR group included a significantly higher proportion of older patients than the other patients (*p* = 0.004). There were no group differences in tumor factors, including ly, v, ne, tumor differentiation, pathological T status (Union for International Cancer Control [UICC] 8th edition), and pathological stage (UICC 8th edition). In patients who did not relapse during adjuvant chemotherapy or within 6 months after surgery, the completion rate of adjuvant chemotherapy was significantly lower in those with low HGS + low LMR than in the other patients (*p* < 0.001). Sarcopenia was more common in the low HGS + low LMR group, a finding consistent with HGS reflecting sarcopenia (*p* < 0.001). There was no difference in the proportion of patients receiving neoadjuvant chemotherapy.

Importantly, patients with low HGS + low LMR were older and had a substantially lower rate of adjuvant chemotherapy completion than other patients. This suggests that the combined marker may partly reflect frailty, reduced physiological reserve, and decreased treatment tolerance, rather than the direct effect of the biomarker combination alone.

## 4. Discussion

In the present exploratory study, LMR and HGS were associated with OS in patients with pancreatic head cancer undergoing PD. The combination of low HGS and low LMR was also associated with poor OS. However, because this was a single-center retrospective study with a small low HGS + low LMR subgroup, the results should be interpreted cautiously and should be considered hypothesis-generating rather than definitive evidence for clinical decision-making.

### 4.1. LMR as a Prognostic Marker

LMR has been used as a prognostic marker for patients with various malignancies [1,6,23], including pancreatic cancer [24,25,26]. LMR represents the ratio of lymphocytes to monocytes. Lymphocytes include tumor-infiltrating lymphocytes, which have antitumor effects and suppress cancer cells. Monocytes differentiate into macrophages, which become tumor-associated macrophages (TAMs) when they infiltrate cancerous tissues, releasing cytokines that promote cancer cell growth and chemokines that suppress tumor immunity. Therefore, low LMR indicates an inadequate immune response against cancer cells, driven by lymphocyte depletion and increased TAM activity. Increased monocyte/macrophage-related activity may promote tumor growth while suppressing antitumor immunity [1,27]. Thus, a low LMR is associated with a poor prognosis.

### 4.2. HGS as a Prognostic Marker

Sarcopenia is related to poor cancer prognosis [12,13,14]. Factors associated with sarcopenia include walking speed, HGS, and skeletal muscle mass. Among them, HGS has the advantages of being simple and quick to perform, inexpensive, noninvasive, objective, and significantly correlated with overall body strength. HGS correlates with muscle mass and quality [28]. Newman et al. reported that muscle strength was more closely associated with survival than muscle mass [29]. Waki et al. also reported that reduced muscle quality was related to poor OS in postoperative patients with gastric cancer [30]. Muscle wasting may reflect severe systemic inflammation associated with tumors with more aggressive biology [31].

Although low HGS is associated with poor prognosis in gastric cancer and cirrhosis [12,13], it has not been adequately studied in pancreatic head cancer. In the present study, patients with low HGS had significantly lower OS rates than those with high HGS.

### 4.3. Underlying Mechanism of HGS and LMR as Prognostic Markers

The biological mechanism underlying the association between the combination of low LMR and low HGS and poor prognosis is likely multifactorial and remains uncertain. Low LMR may reflect impaired antitumor immunity and enhanced tumor-promoting inflammation, including relative lymphopenia and increased monocyte/macrophage-related activity. In contrast, low HGS may reflect sarcopenia, frailty, and reduced physiological reserve. Therefore, the combination of low LMR and low HGS may identify patients with both unfavorable systemic inflammatory/immune status and impaired physiological reserve.

This interpretation is supported by the present finding that patients with low HGS + low LMR were older and were less likely to complete adjuvant chemotherapy. Thus, poorer outcomes in this group may be partly attributable to frailty and reduced treatment tolerance. Previous studies have shown that low skeletal muscle mass and reduced lean body mass are associated with increased chemotherapy toxicity and impaired treatment tolerance [32,33,34]. Although HGS reflects muscle function rather than muscle mass, low HGS may similarly indicate reduced physiological reserve and vulnerability to treatment-related toxicity. The combined marker should therefore not be interpreted as a direct causal biological mechanism, but as a composite preoperative risk marker that captures both tumor-related inflammatory status and host-related vulnerability.

CA19-9 is a well-established prognostic marker in pancreatic cancer and was also associated with poor OS in the present cohort. Recently, Dekker et al. proposed the ABC staging system for localized pancreatic ductal adenocarcinoma, in which tumor anatomy, CA19-9, and performance status at diagnosis were independent prognostic factors [20]. HGS and LMR are not intended to replace CA19-9; rather, they may complement existing preoperative risk stratification by adding information regarding host physical reserve and systemic inflammatory status. In this exploratory cohort, adding CA19-9 to HGS + LMR did not clearly improve discrimination, but this result should be validated in larger cohorts.

We additionally performed an exploratory interaction analysis by adding an HGS × LMR interaction term to the multivariable Cox proportional hazards model. The interaction term was not statistically significant (*p* for interaction = 0.505). Therefore, we interpret the combination of HGS and LMR as a clinically simple composite marker rather than definitive evidence of a synergistic biological effect.

### 4.4. Prehabilitation to Improve Sarcopenia

Recently, the usefulness of prehabilitation as a method for improving sarcopenia has been reported. Prehabilitation refers to preoperative interventions, such as rehabilitation and nutritional therapy, to improve physical function in anticipation of future stressors, such as surgery and chemotherapy [35,36]. It can prevent postoperative complications, promote early independence in physical activity, and improve prognosis [35]. Yamamoto et al. [37] reported that an average of 16 days of exercise and nutritional therapy significantly improved grip strength in 22 preoperative patients with gastric cancer and sarcopenia, with four of the 22 patients showing resolution of sarcopenia.

### 4.5. Neoadjuvant Chemotherapy

Neoadjuvant therapy is increasingly used for borderline resectable pancreatic cancer and selected patients with high-risk resectable disease. However, it should not be described as the current standard of care for all patients with resectable pancreatic cancer [38,39]. In patients undergoing upfront surgery, postoperative adjuvant chemotherapy remains an important component of standard treatment. Therefore, preoperative identification of patients with low HGS + low LMR may be clinically relevant, as these patients may have reduced physiological reserve and lower tolerance to postoperative adjuvant chemotherapy. Future investigations are needed to determine whether prehabilitation or other perioperative interventions can improve patient outcomes in this high-risk group.

### 4.6. Study Limitations

This study has several limitations. First, this was a single-center retrospective study with a relatively small sample size and a short follow-up period. Only 12 patients were included in the low HGS + low LMR group, and therefore the hazard ratio estimates may be unstable. The results should be considered exploratory and hypothesis-generating. Second, external validation was not performed, and the findings may not be generalizable to other cohorts or regions. Third, the cutoff values were derived from the same dataset using ROC analysis and the Youden index, which may have introduced overfitting. Although the LMR cutoff value was similar to previously reported values and the HGS cutoff values were close to commonly used sex-specific criteria for low muscle strength, these cutoff values require external validation in independent cohorts. Fourth, selection bias may have been present because only patients with available preoperative HGS data were included. Fifth, measurement bias cannot be excluded because detailed information regarding examiner training, interobserver reliability, and intraobserver reliability was unavailable. Sixth, residual or unadjusted confounding may remain despite multivariate adjustment, particularly confounding by age, frailty, nutritional status, tumor biology, treatment selection, and adjuvant chemotherapy tolerance. Seventh, several inflammation-based markers are biologically and statistically correlated, and collinearity may have affected model estimation. Finally, this study included only patients with pancreatic head cancer undergoing PD, and the usefulness of low HGS + low LMR should be examined for pancreatic cancer as a whole, including cancers of the pancreatic head, body, and tail.

Despite these limitations, this exploratory study suggests that the combination of HGS and LMR may be useful for preoperative risk stratification in patients with pancreatic head cancer undergoing PD. Larger prospective multicenter studies are needed before this combined marker can be incorporated into routine clinical practice.

## 5. Conclusions

In this single-center exploratory study, the combination of low preoperative LMR and low HGS was associated with poor overall survival in patients with pancreatic head cancer undergoing pancreaticoduodenectomy. This combined marker may help identify high-risk patients before surgery, but the findings should be considered hypothesis-generating because of the small sample size, single-center design, data-driven cutoff values, and lack of external validation. Further prospective multicenter studies are required to confirm its prognostic value and clinical utility.

## Figures and Tables

**Table 1:** Univariate and multivariate analyses of preoperatively measurable factors and overall survival.

*n*			Univariate Analysis		Multivariate Analysis	
			HR (95% CI)	*p* Value	HR (95% CI)	*p* Value
Age (years)	≥74	52	1.65 (0.98–2.79)	0.051		
	<74	53	1			
Sex	Male	59	0.83 (0.49–1.40)	0.490		
	Female	46	1			
CONUT score	>4	21	1.62 (0.85–3.07)	0.140		
	≤4	84	1			
Preoperative CA19-9 level	≥625	12	2.44 (1.18–5.00)	0.015	2.68 (1.25–5.76)	0.011
	<625	93	1		1	
Lymphocyte-to-monocyte ratio	≤3.1	38	1.91 (1.11–3.29)	0.019	2.05 (1.12–3.76)	0.020
	>3.1	67	1		1	
Neutrophil-to-lymphocyte ratio	≥5.3	11	2.14 (0.91–5.02)	0.081		
	<5.3	94	1			
Lymphocyte-to-C-reactive protein ratio	≤3820	32	1.69 (0.97–2.93)	0.063		
	>3820	73	1			
Albumin-to-globulin ratio	≤3.1	39	1.45 (0.83–2.51)	0.185		
	>3.1	66	1			
Platelet-to-lymphocyte ratio	≥198	40	1.23 (0.73–2.11)	0.432		
	<198	65	1			
C-reactive protein-to-albumin ratio	≥0.02	73	0.82 (0.47–1.44)	0.504		
	<0.02	32	1			
Advanced lung cancer inflammation index score	≤0.05	47	0.71 (0.42–1.20)	0.203		
	>0.05	58	1			
Systemic inflammation response index score	≥1652	22	1.60 (0.84–3.06)	0.153		
	<1652	83	1			
Systemic immune-inflammation index score	≥1318	15	1.96 (0.95–4.02)	0.067		
	<1318	90	1			
Body mass index	Low	38	1.25 (0.74–2.13)	0.396		
	High	67	1			
Gait speed	<1.44	84	1.64 (0.91–2.96)	0.102		
	≥1.44	21	1			
Preoperative handgrip strength	Low	33	1.82 (1.04–3.18)	0.035	2.41 (1.29–4.51)	0.006
	High	72	1		1	
Skeletal muscle mass index (L3)	Low	49	0.98 (0.58–1.66)	0.956		
	High	56	1			
Neoadjuvant chemotherapy	Yes	38	1.62 (0.94–2.78)	0.080		
	No	67	1			

Multivariate analysis using the Cox proportional hazards model was performed with variables selected using the stepwise method (*p* = 0.05 for inclusion and removal) among variables that showed a significant difference in univariate analysis. HR, hazard ratio; CONUT, Controlling Nutrition Status; CI, confidence interval; CA19-9, carbohydrate antigen 19-9.

**Table 2:** Univariate and multivariate analyses of clinicopathological factors and overall survival.

*n*			Univariate Analysis		Multivariate Analysis	
			HR (95% CI)	*p* Value	HR (95% CI)	*p* Value
Age (years)	≥74	52	1.65 (0.98–2.79)	0.061		
	<74	53	1			
Sex	Male	59	0.83 (0.49–1.40)	0.490		
	Female	46	1			
Neoadjuvant chemotherapy	Yes	38	1.65 (0.97–2.82)	0.068		
	No	67	1	0.068		
Combined vascular resection	Yes	45	2.50 (1.46–4.26)	<0.001	2.27 (1.29–4.00)	0.004
	No	60	1		1	
Lymphatic invasion (microscopic)	0 or 1	78	1	0.112		
	2 or 3	27	1.57 (0.90–2.77)			
Venous invasion (microscopic)	0 or 1	79	1	<0.001	1	0.032
	2 or 3	26	3.02 (1.74–5.24)		1.94 (1.05–3.56)	
Tumor differentiation	Poor	10	1.57 (0.77–3.20)	0.217		
	Others	95	1			
T status (UICC 8th edition)	1	16	1	0.061		
	2, 3, or 4	89	2.40 (0.95–6.02)			
Lymph node metastasis	Yes	75	2.95 (1.44–6.02)	0.003	2.50 (1.18–5.32)	0.017
	No	30	1		1	
pStage (UICC 8th edition)	I or II	68	1	0.056		
	III or IV	37	1.67 (0.99–2.82)			
Resection curability	0	71	1	0.016	1	0.159
	1 or 2	34	1.91 (1.12–3.24)		1.51 (0.85–2.66)	
Postoperative complications (CD ≥ 3)	Yes	29	1.47 (0.83–2.59)	0.186		
	No	76	1			
Operation time (min)	≥534	47	1.30 (0.78–2.19)	0.317		
	<534	58	1			
Intraoperative bleeding (g)	≥685	51	1.36 (0.81–2.29)	0.247		
	<685	54	1			
Intraoperative transfusion	Yes	28	1.60 (0.91–2.81)	0.099		
	No	77	1			
Preoperative CA19-9 level	≥625	12	2.44 (1.18–5.00)	0.015	1.48 (0.69–3.18)	0.315
	<625	93	1		1	
Low HGS + low LMR	Yes	12	4.66 (1.56–13.9)	<0.001	2.85 (1.06–7.69)	0.038
	No	93	1		1	
Adjuvant chemotherapy	Yes	86	0.60 (0.30–1.19)	0.145		
	No	19	1			
Adjuvant chemotherapy completion *	Yes	48	1	<0.001	1	0.002
	No	57	2.46 (1.44–4.20)		2.53 (1.41–4.50)	

Multivariate analysis using the Cox proportional hazards model was performed with variables selected using the stepwise method (*p* = 0.05 for inclusion and removal) among variables that showed a significant difference in univariate analysis. HR, hazard ratio; UICC, Union for International Cancer Control; CD, Clavien–Dindo classification; CI, confidence interval; CA19-9, carbohydrate antigen 19-9; HGS, handgrip strength; LMR, lymphocyte-to-monocyte ratio. * In patients without recurrence during adjuvant chemotherapy or within 6 months after surgery.

**Table 3:** Characteristics of patients with pancreatic head cancer undergoing pancreaticoduodenectomy.

	Low HGS + Low LMR	Others	*p* Value
	(*n* = 12)	(*n* = 93)	
Age (years)	79 (76–82)	71 (66–78)	0.004
Sex (male), *n* (%)	8 (67)	51 (55)	0.432
Neoadjuvant chemotherapy (yes), *n* (%)	5 (42)	33 (35)	0.677
Combined vascular resection (yes), *n* (%)	7 (58)	38 (41)	0.252
Operative time (min)	501 ± 77	534 ± 122	0.386
Intraoperative blood loss (g)	730 ± 147	685 ± 128	0.506
Intraoperative blood transfusion (yes), *n* (%)	3 (25)	25 (27)	0.889
Postoperative complication (CD ≥ 3) (yes), *n* (%)	5 (42)	24 (26)	0.264
Lymphatic invasion, grade 0/1 vs. 2/3, *n* (%)	9 (75)	69 (74)	0.951
Venous invasion, grade 0/1 vs. 2/3, *n* (%)	8 (67)	71 (76)	0.478
Tumor differentiation, poor vs. others	1 (8.3)	10 (11)	0.110
pT status (UICC 8th edition), T1 vs. T2/3/4	1/11	15/78	0.449
Lymph node metastasis (yes), *n* (%)	9 (75)	66 (71)	0.768
pStage (UICC 8th edition), I/II vs. III/IV	6/6	62/31	0.264
Resection curability, R0 vs. R1/2, *n* (%)	6 (50)	65 (70)	0.178
Adjuvant chemotherapy (yes), *n* (%)	7 (58)	79 (85)	0.041
Adjuvant chemotherapy completion * (yes), *n* (%)	0 (0)	48 (51)	<0.001
Preoperative CA19-9 level (≥625) (yes), *n* (%)	3 (25)	9 (10)	0.158
Sarcopenia (yes), *n* (%)	8 (67)	7 (8)	<0.001

Data are presented as median (interquartile range) for age, mean ± standard deviation for other continuous variables, and number (%) for categorical variables. HGS, handgrip strength; LMR, lymphocyte-to-monocyte ratio; CD, Clavien–Dindo classification; UICC, Union for International Cancer Control; CA19-9, carbohydrate antigen 19-9. * In patients without recurrence during adjuvant chemotherapy or within 6 months after surgery.

## Data Availability

The datasets generated and/or analyzed during the current study are available from the corresponding author on reasonable request.

## Supplementary Materials

The following supporting information can be downloaded at: https://www.mdpi.com/article/10.3390/cancers18142227/s1, Table S1: Spearman correlation coefficients among inflammation- and nutrition-based biomarkers; Table S2: Sensitivity analyses using previously reported lymphocyte-to-monocyte ratio (LMR) cutoff values.

## Abbreviations

AGR	albumin-to-globulin ratio
ALI	advanced lung cancer inflammation index
AUC	area under the curve
CA	carbohydrate antigen
CAR	C-reactive protein-to-albumin ratio
CI	confidence interval
EWGSOP	European Working Group on Sarcopenia in Older People
HGS	handgrip strength
IBPS	inflammation-based prognostic score
LCR	lymphocyte-C-reactive protein ratio
LMR	lymphocyte-to-monocyte ratio
NLR	neutrophil-to-lymphocyte ratio
OS	overall survival
PD	pancreaticoduodenectomy
PLR	platelet-to-lymphocyte ratio
ROC	receiver operating characteristic
SIRI	systemic inflammation response index
SII	systemic immune-inflammation index
SMI	skeletal muscle mass index
