# Supplementary material for: Prognostic Value of the Combined Lymphocyte-to-Monocyte Ratio and Handgrip Strength in Patients with Resected Pancreatic Head Cancer
Source: Cancers (Basel). 2026 Jul 10;18(14):2227. doi: 10.3390/cancers18142227 (PMC13406391; doi:10.3390/cancers18142227)
Supplement: Supplementary file 1 [file cancers-18-02227-s001.zip › cancers-4377303-supplementary.pdf]

Supplementary Materials

Table S1. Spearman correlation coefficients among inflammation- and nutrition-based biomarkers.

Variable	LMR	NLR	PLR	ALI	AGR	SIRI	CAR	SII	LCR
LMR	1.000								
NLR	0.363	1.000							
PLR	0.357	0.210	1.000						
ALI	-0.319	-0.227	-0.425	1.000					
AGR	0.294	0.232	0.077	0.148	1.000				
SIRI	0.538	0.551	0.179	-0.087	0.310	1.000			
CAR	0.283	0.144	0.161	-0.111	0.341	0.188	1.000		
SII	0.486	0.702	0.264	-0.258	0.258	0.592	0.270	1.000	
LCR	0.399	0.292	0.173	-0.230	0.369	0.322	0.694	0.368	1.000

Abbreviations: LMR, lymphocyte-to-monocyte ratio; NLR, neutrophil-to-lymphocyte ratio; PLR, platelet-to-lymphocyte ratio; ALI, advanced lung cancer inflammation index; AGR, albumin-to-globulin ratio; SIRI, systemic inflammation response index; CAR, C-reactive protein-to-albumin ratio; SII, systemic immune-inflammation index; LCR, lymphocyte-to-C-reactive protein ratio.

Table S2. Sensitivity analyses using previously reported lymphocyte-to-monocyte ratio (LMR) cutoff values.

Analysis	LMR cutoff	Kaplan–Meier (Log-rank P)	Multivariable HR (95% CI)	P value
Primary analysis	ROC-derived cutoff	See Table 2	See Table 2	See Table 2
Sensitivity analysis	3.0 (Sierzega et al., 2017)	0.0017	1.91 (1.04–3.50)	0.044
Sensitivity analysis	2.86 (Li et al., 2016)	<0.0001	2.00 (1.01–3.69)	0.032

Primary analysis used the ROC-derived LMR cutoff. Sensitivity analyses were performed using two previously reported LMR cutoff values: 3.0 (Sierzega et al., Ann Surg Oncol 2017) and 2.86 (Li et al., Onco Targets Ther 2016). Hazard ratios were estimated using the same multivariable Cox proportional hazards model as the primary analysis.
